# 1847. A mixed methods approach to understanding the perceived risk of COVID-19 and vaccination perceptions among people living with HIV in Nebraska

**DOI:** 10.1093/ofid/ofad500.1675

**Published:** 2023-11-27

**Authors:** Titilola Labisi, Nada Fadul, Moses New-Aaron, Jason Coleman, Anthony Podany, Keyonna King

**Affiliations:** Kaiser Permanente, Los Angeles, California; University of Nebraska Medical Center, Omaha, Nebraska; University of Nebraska Medical Center, Omaha, Nebraska; University of Nebraska Omaha, Omaha, Nebraska; University of Nebraska Medical Center, Omaha, Nebraska; University of Nebraska Medical Center, Omaha, Nebraska

## Abstract

**Background:**

Despite COVID-19 vaccine benefits, some people with HIV (PWH) might be unvaccinated. Also, whether PWH may have increased risk of severe COVID-19 outcomes due to immunosuppression remain inconclusive. Thus, understanding the perceived risk (PR) of COVID-19 and vaccine perceptions is critical for reduced COVID-19 complication risk and vaccine uptake. We applied a mixed methods approach to understand the PR of COVID-19 and perception of vaccines among PWH.

**Methods:**

We recruited participants from University of Nebraska Medical Center HIV clinic. Eligible criteria included 1) ≥19 years; 2) Nebraska resident; 3) English Language proficiency; 4) HIV diagnosis. Participants (N=100) completed an electronic survey. We operationalized PR using the fear of COVID-19 scale scores. Quantitative data analyses included descriptive statistics, logistic regression and negative binomial. Participants (N=19) were purposefully selected for qualitative phase and completed a semi-structured one-on-one interview. Qualitative data were analyzed using open-ended coding and thematic analysis.

**Results:**

Overall, perceived risk of COVID-19 was moderate (mean 15.3, SD: 7.0) out of 35. Eighty-five percent reported receiving ≥1 vaccine dose and 31% reported a history of COVID-19. Participants aged 19-30 years compared to 31-50 years were less likely to have high perceived COVID-19 risk scores (p= 0.02, RR: 0.70, CI: 0.53-0.93). Similarly, age group 19-30 years was more likely to report COVID-19 infection history (p= 0.03, OR: 1.83, CI: 0.04 – 0.68). The qualitative data reveal that participants’ PR waned over time. However, those with high PR expressed fear of severe COVID-19 complications. Vaccine perceptions were mixed, with lack of trust in the vaccines as main reason for hesitancy. Though not medically confirmed, 4 out of 19 participants interviewed believed they had adverse effects post-COVID-19 vaccination, including stroke, blood clots, and hives.

**Conclusion:**

Our data suggest below national vaccination status among PWH and lack of vaccine trust might be a reason for low vaccination status. Also, younger PWH may have lower PR and higher COVID-19 risk than older PWH. We recommend further research on vaccination uptake, HIV-COVID-19 vaccine interactions, and long-term effects of COVID-19 among PWH.

**Disclosures:**

**All Authors**: No reported disclosures

